# Secretory production of a beta-mannanase and a chitosanase using a *Lactobacillus plantarum* expression system

**DOI:** 10.1186/s12934-016-0481-z

**Published:** 2016-05-12

**Authors:** Suttipong Sak-Ubol, Peenida Namvijitr, Phornsiri Pechsrichuang, Dietmar Haltrich, Thu-Ha Nguyen, Geir Mathiesen, Vincent G. H. Eijsink, Montarop Yamabhai

**Affiliations:** Molecular Biotechnology Laboratory, School of Biotechnology, Institute of Agricultural Technology, Suranaree University of Technology, Nakhon Ratchasima, Thailand; Food Biotechnology Laboratory, Department of Food Science and Technology, BOKU-University of Natural Resources and Life Sciences, Vienna, Austria; Department of Chemistry, Biotechnology and Food Science, Norwegian University of Life Sciences (NMBU), Ås, Norway

**Keywords:** β-Mannanase, Chitosanase, *L. plantarum*, pSIP, Alanine racemase, Secretion, Food-grade, *Bacillus*, Signal peptide, OmpA

## Abstract

**Background:**

Heterologous production of hydrolytic enzymes is important for green and white biotechnology since these enzymes serve as efficient biocatalysts for the conversion of a wide variety of raw materials into value-added products. Lactic acid bacteria are interesting cell factories for the expression of hydrolytic enzymes as many of them are generally recognized as safe and require only a simple cultivation process. We are studying a potentially food-grade expression system for secretion of hydrolytic enzymes into the culture medium, since this enables easy harvesting and purification, while allowing direct use of the enzymes in food applications.

**Results:**

We studied overexpression of a chitosanase (CsnA) and a β-mannanase (ManB), from *Bacillus licheniformis* and *Bacillus subtilis*, respectively, in *Lactobacillus plantarum*, using the pSIP system for inducible expression. The enzymes were over-expressed in three forms: without a signal peptide, with their natural signal peptide and with the well-known OmpA signal peptide from *Escherichia coli.* The total production levels and secretion efficiencies of CsnA and ManB were highest when using the native signal peptides, and both were reduced considerably when using the OmpA signal. At 20 h after induction with 12.5 ng/mL of inducing peptide in MRS media containing 20 g/L glucose, the yields and secretion efficiencies of the proteins with their native signal peptides were 50 kU/L and 84 % for ManB, and 79 kU/L and 56 % for CsnA, respectively. In addition, to avoid using antibiotics, the erythromycin resistance gene was replaced on the expression plasmid with the alanine racemase (*alr*) gene, which led to comparable levels of protein production and secretion efficiency in a suitable, *alr*-deficient *L. plantarum* host.

**Conclusions:**

ManB and CsnA were efficiently produced and secreted in *L. plantarum* using pSIP-based expression vectors containing either an erythromycin resistance or the *alr* gene as selection marker.

## Background

Heterologous production of hydrolytic enzymes is important for green and white biotechnology since such enzymes serve as green industrial biocatalysts for the conversion of biomass into value-added products [1]. Lactic acid bacteria (LAB) are interesting hosts for the production of such enzymes because many of these bacteria are generally recognized as safe (GRAS), carry the qualified presumption of safety (QPS) status, and are easy to cultivate [2]. While LAB may not be the most efficient cell factories, their safety and food-grade status make them particularly attractive for producing enzymes that are to be used in e.g. food processing. One attractive host is *Lactobacillus plantarum*, as it had been widely used for foods, and hence is food-grade and even considered a probiotic [3] with potential benefits to human health [4]. To facilitate downstream processing in large-scale biotechnological applications, secretion of the over-expressed enzymes into the culture medium is desirable [5]. Therefore, lactobacillal expression systems based on the so-called pSIP vectors [6, 7] have been developed recently for the efficient secretion of heterologous proteins in *L. plantarum* [8].

In the present study we selected two extracellular, hydrolytic enzymes from *Bacillus*, a β-mannanase from *Bacillus licheniformis* (*Bl*ManB) and a chitosanase from *Bacillus subtilis* (*Bs*CsnA), to study secretory production in *L. plantarum*. Both enzymes are of interest for biotechnological applications, namely the conversion of hemicelluloses (mannans) and chitosan into manno-oligosaccharides (MOS) [9] and chito-oligosaccharides (CHOS) [10], respectively. For comparative purposes, these enzymes were overproduced in three forms: with no signal peptide, with their native (*Bacillus*) signal peptide, and with a signal peptide derived from the *Escherichia coli* OmpA protein. In addition, we compared two different selection markers, one based on antibiotic (erythromycin) resistance, and the other based on complementation selection using alanine racemase (*alr*). The engineered production strains were evaluated in terms of enzyme yields and secretion efficiencies.

## Results

### Construction of expression vectors

**Fig. 1:**
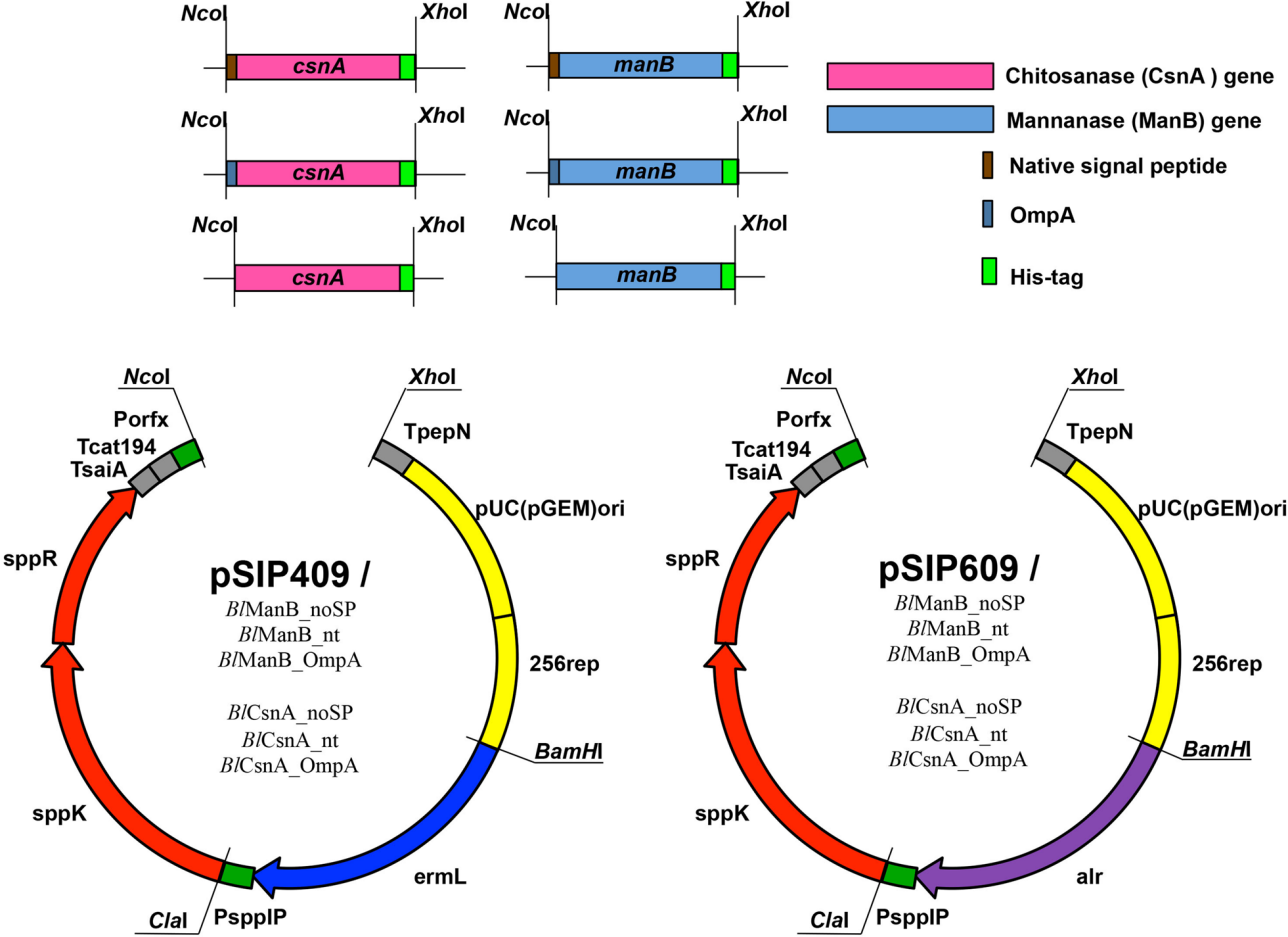
Vector construction. Both pSIP409 and pSIP609 vectors were used for expression of *B. licheniformis* β-mannanase (*Bl*ManB) and *B. subtilis* chitosanase (*Bs*CsnA). Enzyme expression was under the control of the P_orfx_ promoter (also known as P_sppQ_) [32], which can be induced by the 19-residue peptide pheromone IP-673. The vectors contain an erythromycin resistance (*erm* ^*R*^) or an alanine racemase (*alr*) gene as selection marker, for pSIP409 and pSIP609, respectively. Polyhistidine tags were incorporated C-terminally to facilitate one-step affinity purification. The 256_rep_ replicon allows DNA replication in *L. plantarum*. Each enzyme was cloned in three forms, two of which contain a signal peptide for secretion (native or OmpA). The genes marked in *red* constitute the two-component system needed for peptide-pheromone driven induction; the *grey areas* marked with a T are terminator sequences

Genes encoding a mannan endo-1,4-β-mannosidase or 1,4-β-D-mannanase (EC 3.2.1.78), commonly named β-mannanase (ManB), from *B. licheniformis* strain DSM13 [11] and a chitosan *N*-acetylglucosaminohydrolase or chitosanase (EC 3.2.1.132) (CsnA, previously termed Csn) from *B. subtilis* strain 168 [12] were initially cloned into pSIP409 [7]. Subsequently, the erythromycin resistance gene (*erm*^*R*^) in the pSIP409-based constructs was replaced with the alanine racemase gene (*alr*) [13, 14] to generate food-grade pSIP609 expression vectors, as shown in Fig. 1. Each gene was cloned in three forms: with no signal peptide (*Bl*ManB_noSP and *Bs*CsnA_noSP), with their native signal peptides (*Bl*ManB_nt and *Bs*CsnA_nt) or with the *E. coli* OmpA signal peptide (*Bl*ManB_OmpA and *Bs*CsnA_OmpA). The ability of the signal peptides to direct the secretion of these enzymes *in E. coli* has previously been reported [12, 15].

### Expression and secretion of Bl*ManB and* Bs*CsnA* in *L. plantarum*

**Fig. 2:**
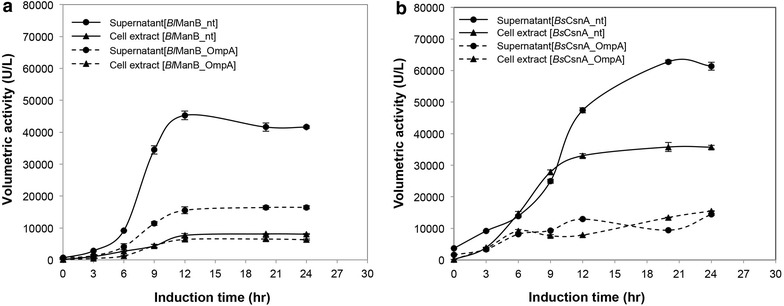
Production and secretion of *B. licheniformis* β-mannanase (*Bl*ManB, **a**) and *B. subtilis* chitosanase (*Bs*CsnA, **b**) using pSIP409-type constructs containing the native or the OmpA signal peptide. The recombinant *L. plantarum* strains were batch-cultured in 3-L vessels with pH control at pH 6.5. After harvesting at various time points, volumetric enzyme activities in both the culture supernatant and the cell lysate were determined as described in the “Methods” section. Data given are the average of two independent experiments ± their standard deviation. The *lines* connecting the *points* are drawn for illustration purposes only. Key data, supplemented with data for the constructs without signal peptide are summarized in Table 1

Recombinant strains of *L. plantarum* were grown in 3-L fermenters using MRS medium as described in the “Methods” section. The pH of the culture was controlled at 6.5 using a 3 N NaOH solution. Enzyme activities were determined in both culture supernatants and cell lysates to calculate volumetric activities at different time points during the cultivations (Fig. 2; Table 1). Both *Bacillus* enzymes were secreted when using either of the two signal peptides, yet using the native signal peptides resulted in both the highest total production levels and the highest secretion efficiencies. While these data show that the OmpA signal peptide from Gram-negative *E. coli* does function in *Lactobacillus*, they also indicate that signal peptides from Gram-positive bacilli work better.

**Table 1:** Yields and secretion efficiency for production of *Bl*ManB and *Bs*CsnA in* L*. *plantarum* WCFS1 harboring various expression constructs based on the pSIP409 vector

Enzyme	Type of SP	Volumetric activity (per liter)			% Secretion efficiency	Specific activity (U/mg)	
		Broth	Cell extract	Total		Broth	Cell extract
ManB	Native SP	42 ± 1.3 kU	8 ± 0.2 kU	50 ± 1.5 kU	83.7	139	47
	OmpA SP	16 ± 0.6 kU	7 ± 0.3 kU	23 ± 0.9 kU	71.6	84	39
	No SP	5 ± 0.2 kU	20 ± 0.9 kU	25 ± 1.1 kU	19.0	–	92
CsnA	Native SP	63 ± 0.5 kU	36 ± 1.4 kU	99 ± 1.9 kU	63.7	195	168
	OmpA SP	9 ± 0.03 kU	13 ± 0.1 kU	22 ± 0.13 kU	41.2	90	97
	No SP	1 ± 0.05 kU	15 ± 0.7 kU	16 ± 0.8 kU	6.7	–	65

The data were obtained from the culture harvested at 24 h after induction as described in “Methods” section. Data given are the average of two independent experiments ± their standard deviation. Percentage secretion efficiency was calculated by dividing the enzyme activity in the culture broth by the total enzyme activity (broth + cell extract) ×100

The genome of *L. plantarum* WCFS1 does not encode any known β-mannanase or chitosanase (www.cazy.org; [16]). Since mannanase activity in certain *L. plantarum* strains has however been reported [17], we checked the intrinsic β-mannanase and chitosanase activities in *L. plantarum* WCFS1 and its *alr* derivative TGL02, which were used as host strains in this study. Using the same fermentation and analytical procedures as above, we could not detect any chitosanase activity in the cell lysate or the culture supernatant of either strain. As for β-mannanase activity, we did not detect any activity in the lysates, while a trace amount of β-mannanase activity was detected in culture supernatants (about 150 units/L; i.e. less than 1 % of typical values shown in Fig. 2). Thus, the enzyme activities reported and discussed in this study are essentially devoid of background activity from the host bacterium.

### Purification and analysis of secreted Bl*ManB* and Bs*CsnA*

**Fig. 3:**
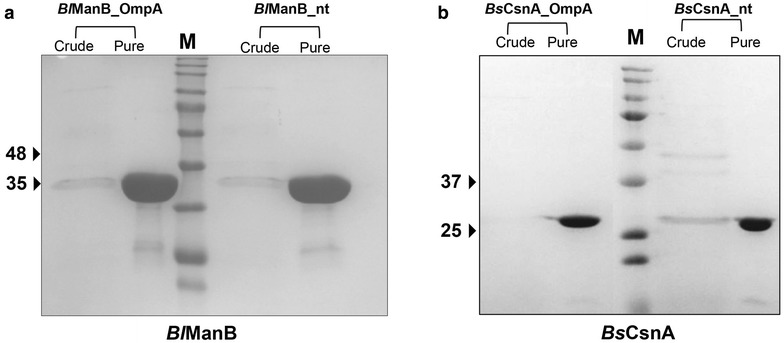
SDS-PAGE analysis of culture supernatants and purified *Bl*ManB and *Bs*CsnA. The Coomassie-stained gels illustrate the purification of *B. licheniformis* ManB (**a**) and *B. subtilis* CsnA (**b**) from culture supernatants. For crude culture supernatants, 20 µL of sample was loaded, whereas for the purified enzymes a total of 20 and 5 µg protein of ManB and CsnA samples, respectively, were loaded. *M* indicates the Kaleidoscope protein standard (Bio-Rad). Detailed information on the protein contents and enzyme activities in these samples are provided in Table 1

**Fig. 4:**
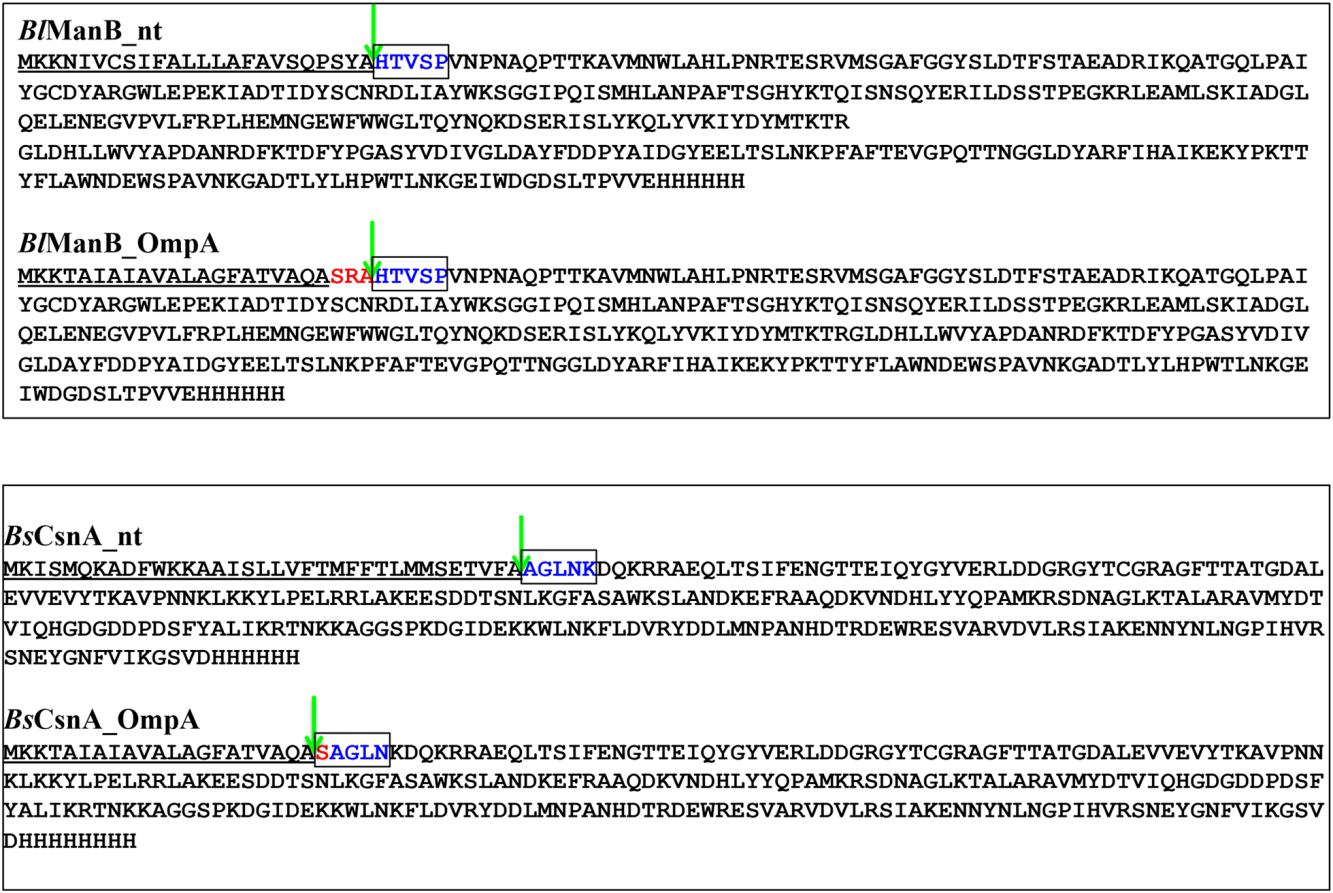
Signal peptide cleavage sites. N-terminal sequence analysis of purified secreted proteins was performed by Edman degradation. The sequences of the native *Bacillus* signal peptides and the *E. coli* OmpA signal peptide are *underlined*. *Arrows* indicate the cleavage sites as deduced from sequence analysis. The first five amino acids obtained by Edman degradation are *colored* and *boxed*. Amino acids in *red* indicate extra amino acids that were introduced during genetic engineering of the fusions between the *Bacillus* enzymes and the *E. coli* OmpA signal peptide

ManB and CsnA produced using the native or the *E. coli* signal peptide were purified from culture broths and cell lysates by single-step immobilized metal affinity chromatography (IMAC) and analyzed by SDS-PAGE. Figure 3 shows that all recombinant enzymes could be purified to a high degree of homogeneity. Routinely, 12 or 9 mg of purified ManB, and 25 or 12 mg of purified Csn, for constructs containing the native or the *E. coli* signal peptide, respectively, could be obtained from 1 L of culture medium. These rather low purification yields in the range of 20–30 % result from strict pooling of only the purest and most active fractions from the IMAC step. The specific activities of purified ManB and CsnA were approximately 1800 and 800 U/mg, respectively, for all samples of purified protein. Determination of the N-terminal sequences of the purified proteins by Edman degradation (Fig. 4) showed that the native *Bacillus* signal peptides of both ManB and CsnA were correctly processed by the *L. plantarum* secretion machinery. For technical reasons the two OmpA signal peptides contained minor variations (Fig. 4), which led to slight variations in processing. For ManB, the protein secreted with OmpA had the same N-terminal sequence as the protein secreted with its native signal peptide, while CsnA secreted with OmpA contained an additional serine residue at its N-terminus.

### Expression of Bl*ManB* and Bs*CsnA* using a food-grade vector system

**Fig. 5:**
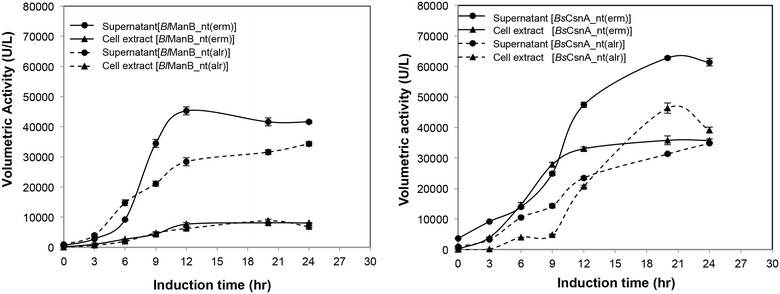
Effects of the selection marker on production of *Bl*ManB (**a**) and *Bs*CsnA (**b**). The figures show the volumetric activities in culture supernatants and cell lysates of *L. plantarum* strains harboring pSIP409-based or pSIP609-based expression vectors, which are based on using *erm* ^*R*^ or *alr* as selection marker, respectively. All conditions were as in Fig. 2. Data given are the average of two independent experiments ± their standard deviation. The *lines* connecting the *points* are drawn for illustration purposes only

**Fig. 6:**
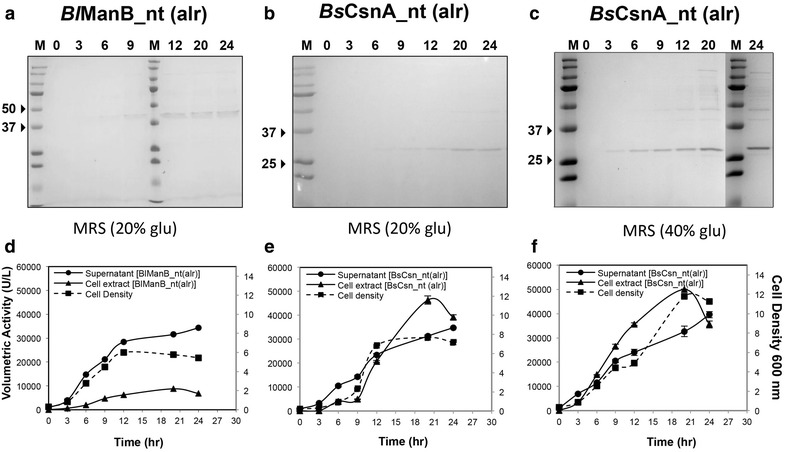
Production of *Bl*ManB and *Bs*CsnA using a food-grade expression system. *L. plantarum* TLG02 strains, harboring pSIP609/*Bl*ManB_nt [(BlManB_nt(alr)] or pSIP609/*Bs*CsnA_nt [(BsCsn_nt (alr)], were cultured in 3-L batch fermentations in MRS medium, containing 20 or 40 g/L glucose as indicated. The *upper panels* show Coomassie-stained SDS-PAGE gels with culture supernatants collected at various time points (20 µL of sample per lane). The *bottom panels* show the time course of the cultivation corresponding to the SDS-PAGE samples. The pH of the batch cultivation was kept constant at 6.5. Note that doubling the amount of glucose led to a doubling of OD_600_, but only to a small increase in enzyme production levels (Table 2)

To demonstrate the applicability of the secretory production of recombinant ManB and CsnA in the food biotechnology industry, the antibiotic selection marker in the pSIP409/*Bl*ManB and pSIP409/*Bs*CsnA expression vectors was replaced with the alanine racemase (*alr*) gene [13, 18]. Based on the results of the experiments described above, only constructs with native signal peptides were used. The resulting expression plasmids, pSIP609/*Bl*ManB and pSIP609/*Bs*CsnA (Fig. 1) were transformed into *L. plantarum* strain TLG02, which is an d-alanine auxotroph [14]. The cultivation conditions were similar to those used for strains harboring pSIP409-derived vectors, except that no antibiotic was added in the culture media. Figure 5 shows a comparison of the volumetric activities of *Bl*ManB and *Bs*CsnA using either *erm*^*R*^ or *alr* as selection marker, at various time points after induction. For both enzymes, production levels were higher for the constructs with the *erm*^*R*^ selection marker, and this was almost exclusively due to higher levels of secreted enzymes. The level of intracellular enzyme activities was hardly affected by the change in the resistance marker, and, consequently, the calculated secretion efficiencies were lower when using the *alr*-based vectors. A summary of total volumetric activity and secretion efficiency obtained using *alr* selection is provided in Table 2. The expression and secretion of recombinant *Bl*ManB and *Bs*CsnA with the food-grade *L. plantarum* expression system could also be detected by SDS-PAGE analysis of culture supernatants (Fig. 6a–c), showing strong enzyme bands. For the CsnA-producing strain, we assessed the effect of increased glucose supply; as expected, higher cell densities were obtained (Fig. 6), but total enzyme production was only marginally increased and the secretion efficiency went slightly down (Table 2).

**Table 2:** Yields and secretion efficiency for production of *Bl*ManB_nt and BsCsnA_nt by *L.* *plantarum* TLG02 (d-alanine auxotroph), measured 20 h after induction

Enzyme	Glucose (g/L)	Volumetric activity (per liter)			% Secretion efficiency
		Broth (kU)	Cell extract (kU)	Total (kU)	
ManB	20	31 ± 0.8	9 ± 0.2	40 ± 1	78.2
CsnA	20	31 ± 0.5	38 ± 0.7	69 ± 1.2	45.1
CsnA	40	33 ± 0.1	50 ± 2.2	83 ± 2.3	39.5

Data given are the average of two independent experiments ± their standard deviation

## Discussion

In this study we show that a β-mannanase (*Bl*ManB) from *B*. *licheniformis* as well as a chitosanase (*Bs*CsnA) from *B. subtilis* can be expressed and secreted efficiently in *L. plantarum* using the pSIP expression system. Both enzymes were produced and secreted at high levels compared to the levels previously obtained using *E. coli* expression systems [11, 12], also when using a potentially food-grade vector system that does not depend on the use of an antibiotic resistance selection marker. When estimated from the specific activities of the purified enzymes (1800 U/mg for ManB and 800 U/mg for CsnA), total yields of recombinant proteins were ~28 mg/L medium for ManB produced with its native signal and a pSIP409-based vector, and ~127 mg/L of recombinant CsnA, again when using the native signal and a pSIP409-derived vector, 24 h after induction (Table 1). The amount of secreted, extracellular recombinant protein for these constructs was ~23 mg/L medium for ManB and ~79 mg/L for CsnA, showing again the efficient secretion of these recombinant enzymes. Replacement of the selection marker from *erm*^*R*^ to *alr* led to slightly lower expression levels (~22 mg of total ManB and ~17 mg of secreted ManB per L medium; ~86 mg of total CsnA and ~39 mg of secreted CsnA per L medium; calculated from data given in Table 2 and the specific activities of the purified enzymes). The lower overall expression levels with the *alr* marker could reflect different plasmid copy numbers due to different selective pressures. Indeed, a previous comparative study on intracellular expression of β-galactosidases showed that *alr* selection led to lower plasmid copy numbers and slightly lower protein production levels [14]. Since the difference in expression level primarily concerned the secreted fraction of the produced protein, one could also speculate about connections between cell wall metabolism (which is effected by the *alr* mutation) and protein secretion.

It should be noted that further optimization can be done to obtain higher production levels with the various expression set-ups developed here. Possible variables include the glucose concentration and the glucose-feeding regime, the amount of inducing peptide added as well as the time point of induction, the cultivation temperature, and the harvesting time [19].

The potential of using pSIP vectors for both intracellular protein production and protein secretion has been previously reported for model enzymes such as nuclease (NucA) and amylase (AmyA) [20] as well as other enzymes including β-glycosidases [21], oxalate decarboxylase [22, 23], cellulases and xylanases [24], and L-arabinose and D-xylose isomerases [25]. Most of these studies used pSIP vectors with the *erm* antibiotic selection marker, with the exception of studies on the expression of intracellular β-galactosidase [14], and L-arabinose and D-xylose isomerase [25], in which the food-grade *alr* selection marker was used. The present results underpin the usefulness of the *alr* selection marker for food-grade applications.

Notably, since the pSIP vector is a modular plasmid, existing constructs could easily be modified to suit desired purposes [7]. For example, the p256 replicon, which only functions in a limited range of lactobacilli [26] could be exchanged to allow broader host range. This could be useful because lactobacilli have different properties when it comes to e.g. probiotic activity, acid production, production of antimicrobial compounds such as bacteriocins, and the ability to interact with the human mucosa [5]. Notably, if the *alr* selection marker is to be used, application of pSIP vectors in other lactobacilli would require the engineering of d-alanine auxotrophs for each host strain [14].

The native signal peptides of the two *Bacillu*s hydrolytic enzymes functioned well in that they gave good secretion efficiencies, comparable to those obtained with the better performing signal peptides of *L. plantarum* itself, as assessed in previous genome-wide signal peptide-screening studies [20]. Since *Bacillus* also is a Gram-positive bacterium, it is not surprising that the native *Bacillus* signal peptides were efficient in directing secretion of heterologous proteins in Gram-positive *L. plantarum*. The *Bacillus* ManB signal peptide seemed particularly efficient reaching secretion efficiencies in the order of 80 %, and should perhaps be considered for use in secretion of other heterologous proteins in *L. plantarum*.

The data presented above show that the choice of the signal peptide not only affects the secretion efficiency but also the total expression level. This has been observed before [8, 20] and is likely due to effects of the 5′ part of the gene sequence and/or the amino acid sequence of the N-terminal part of the translated protein on overall translation efficiency [27]. The level of protein production apparently determines the secretion efficiency as well. It is likely that when the protein is expressed above a certain critical amount, saturation of the secretion machinery will occur [28, 29]. The latter could be the case for *Bs*CsnA, which is expressed at more than threefold higher levels and even secreted in higher amounts, but with a lower overall secretion efficiency compared to *Bl*ManB (Table 1). Possibly, enzyme size also plays a role; the better produced *Bs*CsnA (~30 kDa) is smaller than *Bl*ManB (~41 kDa).

## Conclusions

An efficient expression and secretion system for food-grade production of a β-mannanase (ManB) and a chitosanase (CsnA) in *L. plantarum* has been established. Our results indicate that native *Bacillus* signal peptides can be used for efficient expression and secretion of heterologous proteins in *L. plantarum*, providing an alternative for homologous, and, in a sense, more “food-grade” lactobacillal signal peptides. The modular pSIP vectors and the *alr* selection marker provide useful tools for the expression of heterologous proteins in *L. plantarum*.

## Methods

### Materials

Plasmids CsnNative-pMY202 and CsnOmpA-pMY202 containing the chitosanase (*csnA*) gene from *B. subtilis* with its native or the OmpA signal peptide, respectively, and a C-terminal His-tag [12] were used as templates for amplification of chitosanase constructs in this study. The plasmid manBOmpA-pMY202 [11] was used as template for amplification of the *B. licheniformismanB* gene containing the OmpA signal peptide. *B. licheniformis* DSM13 (NCBI accession number NC006322.1) genomic DNA was used as a template for amplifying a *manB* variant containing the native signal peptide. *E. coli* Top10 and MB1259 cells were used for molecular cloning with the pSIP409 and pSIP609 vectors, respectively. *L. plantarum* strains WCFS1 [30] and TGL02 [14] were used for expression studies with pSIP409 and pSIP609, respectively.

### Constructs based on pSIP409 (antibiotic selection)

Because of the internal *Nco*I restriction site in the *manB* and *csn* genes, preventing straightforward restriction cloning, a sticky PCR-based method [31] was used to insert the two genes in between the *Nco*I and *Xho*I sites of the pSIP409 expression vector. All oligonucleotide primers used in this study are listed in Table 3 and were designed to allow cloning into the *Nco*I and *Xho*I restriction sites of the pSIP plasmids. Generation of sticky PCR products was performed as previously described with some modification [31]. The PCR reaction mixture (total volume of 50 µL) contained 10 µM of each primer, 10 mM dNTPs, 1.25 units of *Pfu* DNA Polymerase and 10× *Pfu* Buffer with MgSO_4_, provided by the manufacturer (Promega, Madison, USA). The amplification conditions for the *csn* gene were as follows: initial denaturation at 95 °C for 2 min, followed by 30 cycles of 95 °C for 45 s, annealing at 55 °C for 30 s, and extension at 72 °C for 2 min, followed by a final extension step at 72 °C for 10 min. The amplification conditions for the *manB* gene were as follows: initial denaturation at 95 °C for 2 min, followed by 30 cycles of 95 °C for 45 s, annealing at 50 °C for 30 s, and extension at 72 °C for 2.5 min, followed by a final extension step at 72 °C for 10 min. All PCR products and vectors were separated using 1 % agarose gels in 1× TAE containing 0.2 µg/mL of ethidium bromide and visualized under a UV transilluminator. The PCR products and vectors were purified using the Wizard^®^ SV gel and PCR Clean-Up system (Promega, Madison, USA). For constructing complete gene inserts, approximately equal amounts of PCR products were mixed together in a PCR tube and heated at 95 °C for 5 min, and then the denatured products were briefly mixed in a vortex mixer. The re-annealing was done in a thermal cycler machine by reducing the temperature slowly from 95 to 25 °C. The total time for re-annealing was in the order of 2–3 h.

**Table 3:** Oligonucleotide primers used in this study

Construct	Name	Sequence (5′–3′)
*Bs*CsnA_nt	B.subCsnfwNcoIlong	CATGAAAATCAGTATGCAAAAAGCAGATTTTTGG
	B.subCsnfwNcoIshort	AAAATCAGTATGCAAAAAGCAGATTTTTGG
	6HisXhoIrvlong	TCGAGTCAATGGTGATGGTGATGGTG
	6HisXhoIrvshort	GTCAATGGTGATGGTGATGGTG
*Bs*Csn_OmpA & *Bl*ManB_OmpA	FlagNcoIfwlong	CATGAAAAAGACAGCTATCGCGATTG
	FlagNcoIfwshort	AAAAAGACAGCTATCGCGATTG
	6HisXhoIrvlong	TCGAGTCAATGGTGATGGTGATGGTG
	6HisXhoIrvshort	GTCAATGGTGATGGTGATGGTG
*Bl*ManB_nt	B.liManBfwNcoIlong	CTAGAAAAAAAACATCGTTTGTTCAATCT
	B.liManBfwNcoIshort	AAAAAAAACATCGTTTGTTCAATCTTCG
	B.liManB6HisXhoIlong	TCGAGTCAATGGTGATGGTGTTCCACGACAGGCGTCA
	B.liManB6HisXhoIshort	GTCAATGGTGATGGTGTTCCACGACAGGCGTCA
*Bs*CsnA_noSP	B.SubMatCsnNcoIFwlong	CATGGCGGGACTGAATAAAGATC
	B.SubMatCsnNcoIFwshort	GCGGGACTGAATAAAGATCAAAAGC
	6HisXhoIrvlong	TCGAGTCAATGGTGATGGTGATGGTG
	6HisXhoIrvshort	GTCAATGGTGATGGTGATGGTG
*Bl*ManB_noSP	B.LimanBMatfwNcoIlong	CATGGCACACACCGTTTCTCCGGTG
	B.LimanBMatfwNcoIshort	GCACACACCGTTTCTCCGGTG
	6HisXhoIrvlong	TCGAGTCAATGGTGATGGTGATGGTG
	6HisXhoIrvshort	GTCAATGGTGATGGTGATGGTG

The primers used for the construction of each construct by sticky-PCR based method are listed. Note that primers 6HisXhoIrvlong and 6HisXhoIrvshort were used for every construct except for *Bl*ManB_nt. The long and short primer pairs were used to generate sticky 5′ or 3′ ends as previously described [28]

Plasmid pSIP409 [32] was digested with *Nco*I and *Xho*I, and gel-purified before being used in ligation reactions. In these reactions the molar ratio of linearized vector to re-annealed insert was approximately 1:15. The amount of linearized vector used for each ligation reaction was 50–100 ng. Ligations were performed for 16 h at 16 °C in the presence of T4 DNA ligase in a final volume of 25 µL. T4 DNA ligase was heat-inactivated (65 °C for 15 min) before transformation of the ligation mixtures into competent *E. coli* TOP10 cell. Transformants were selected on LB agar containing 800 µg/mL erythromycin; plates were incubated at 37 °C for 16 h.

### Construction of food-grade expression vectors (pSIP609 series)

Food-grade expression vectors for the production of recombinant *Bl*ManB and *Bs*CsnA were constructed by replacing the *erm*^*R*^ gene with the *alr* obtained from pSIP609 [14]. The *erm*^*R*^ genes in pSIP409/*Bs*CsnA_nt, pSIP409/*Bs*CsnA_OmpA, pSIP409/*Bl*ManB_nt and pSIP409/*Bl*ManB_OmpA were exchanged with the *alr* gene using the restriction sites *BamH*I and *Cla*I, resulting in pSIP609/*Bs*CsnA_nt, pSIP609/*Bs*CsnA_OmpA, pSIP609/*Bl*ManB_nt and pSIP609/*Bl*ManB_OmpA, respectively. A list of all plasmids used in this study is shown in Table 4.

**Table 4:** Plasmids used in this study

Plasmid	Description	Reference
pSIP409gusA	*erm*, pSIP401 derivative, *gusA* controlled by P*sppQ*	[7]
pSIP609gusA	pSIP409 derivative, *erm* replaced by *alr*	[34]
CsnNative-pMY202	pFLAG-CTS derivative, *csn_nt* controlled by tac	[12]
CsnOmpA-pMY202	pFLAG-CTS derivative, *csn_OmpA* controlled by tac	[12]
manBOmpA-pMY202	pFLAG-CTS derivative, *manB_ompA* controlled by tac	[11]
pSIP409/*Bs*CsnA_nt	*erm*, pSIP409 derivative, *csn_nt* controlled by P*sppQ*	This work
pSIP409/*Bs*CsnA_OmpA	*erm*, pSIP409 derivative, *csn_OmpA* controlled by P*sppQ*	This work
pSIP409/*Bs*CsnA_noSP	*erm*, pSIP409 derivative, *csn* controlled by P*sppQ*	This work
pSIP609/*Bs*CsnA_nt	*alr*, pSIP409 derivative, *csn_nt* controlled by P*sppQ, erm* replaced by *alr*	This work
pSIP409/*Bl*ManB_nt	*erm*, pSIP409 derivative, *manB_nt* controlled by P*sppQ*	This work
pSIP409/*Bl*ManB_OmpA	*erm*, pSIP409 derivative, *manB_OmpA* controlled by P*sppQ*	This work
pSIP409/*Bl*ManB_noSP	*erm*, pSIP409 derivative, *manB* controlled by P*sppQ*	This work
pSIP609*Bl*ManB_nt	*alr*, pSIP409 derivative, *manB_nt* controlled by P*sppQ, erm* replaced by *alr*	This work

### Transformation of *L. plantarum*

*Lactobacillus plantarum* competent cells were prepared and transformed by electroporation as previously described [33]. To transform the competent cells, 2–5 µg of plasmid DNA was added to 40 µL of electrocompetent cells. The mixture was then transferred to chilled cuvettes. After drying and cleaning the outside of the cuvette it was placed into the electroporator, after which the cells were electroporated at 1.5 kV, followed by incubation on ice for 2 min. After adding 500 µL of MRS medium containing 0.5 M glucose and 0.1 M MgCl_2_ the cells were transferred to a clean 1.5 mL tube and incubated at 30 °C without agitation for 1–2 h. Finally, the cells were plated out on MRS agar plates containing 200 µg/mL of erythromycin (for pSIP409-type vectors transformed to *L. plantarum* WCFS1) or containing no antibiotics (for pSIP609-type vectors transformed to *L. plantarum* TGL02). Colonies appeared after incubation at 37 °C for 16 h.

### *Expression of* Bl*ManB and* Bs*CsnA in L. plantarum*

Batch fermentations with pH control were carried out in 3-L MRS medium using a BIOSTAT B plus bioreactor (Sartorius, Germany). Recombinant *L. plantarum* strains were taken from a glycerol stock stored at −80 °C, re-streaked on appropriate MRS plates (with or without antibiotic, depending on the *L. plantarum* strain; see above) and grown overnight at 37 °C. Five to ten colonies were picked and grown in 5 mL MRS broth overnight, then sub-cultured into two flasks of 100 mL of MRS, and cultivated at 37 °C without shaking for 18–24 h. The two overnight cultures were pooled together, mixed well and after measuring the cell density at 600 nm (Ultrospec 2000, Pharmacia biotech, UK) they were used to inoculate 3 L of MRS medium to an OD_600_ of ~0.1. After incubation at 30 °C with 100 rpm agitation under anaerobic condition to an OD_600_ of ~0.3, the cultures were induced with 12.5 ng/mL of IP-673 (amino acid sequence of IP-673 is Met-Ala-Gly-Asn-Ser–Ser-Asn-Phe-Ile-His-Lys-Ile-Lys-Gln-Ile-Phe-Thr-His-Arg; [34]). During further cultivation (30 °C with 100 rpm), the pH was controlled at pH 6.5 using 3.0 M sodium hydroxide. To monitor enzyme production, 40–50 mL of culture broth were harvested at 0, 3, 6, 9, 12, 18, 20, and 24 h after induction. The cells and culture supernatant were separated by centrifugation at 4000 rpm for 15 min at 4 °C (swing angle rotor, Centrifuge 5804, Eppendorf, Belgium), after which the cells were washed twice with lysis buffer (20 mM Tris–HCl, 150 mM NaCl, pH 8.0), and re-suspended in 3–4 mL of the same buffer. The cells were broken using a sonicator (Vibra-Cell Sonicator, Sonics & Materials, Inc, USA) at 25 % amplitude, pulse 5 s, 3 min for 2 rounds on ice. The cell lysate fraction was collected by centrifugation at 13,000 rpm, 4 °C for 45 min (Thermo Scientific, USA).

To measure the enzyme activity in culture supernatants, 3–5 mL of culture supernatant containing secreted enzymes were dialyzed with 10 mM Tris–HCl buffer, pH 8.0 with stirring at 250 rpm, at 4 °C for 8–12 h, using the snake skin dialysis tubing, 10 kDa kit (Thermo scientific, USA). The dialyzed fraction of approx. 4–7 mL was collected and kept on ice for no longer than 6 h before the enzyme activity was determined.

### Enzyme activity assay

ManB and CsnA activities in both lysates and supernatants were determined using the DNS method as previously described [11, 12]. For *Bl*ManB, an appropriately diluted enzyme solution (0.1 mL) was incubated with 0.9 mL of pre-heated 0.5 % (w/v) locust bean gum (dissolved in 50 mM sodium citrate buffer, pH 6.0) at 50 °C for exactly 5 min, with mixing at 800 rpm. The amount of reducing sugars liberated in the enzyme reaction was assayed by mixing 100 μL of the reaction mixture with 100 μL DNS solution, followed by heating at 100 °C for 20 min, cooling on ice, and dilution with 300 μL of de-ionized water, before measuring the absorbance at 540 nm, using 1–5 µmol/mL of d-mannose as standards. The reactions were done in triplicate and we report mean values together with their standard deviation. The substrate solution was prepared by suspending 0.5 % (w/v) locust bean in 50 mM sodium citrate buffer, pH 6.0. The suspension was then dissolved at 80 °C, using hot plate stirrer at 200 rpm. (RCT CL, IKA Laboratory, Germany), followed by heating to the boiling point, cooled and stored overnight with continuous stirring. After that insoluble material was removed by centrifugation.

For *Bs*CsnA, the reaction mixture consisted of 40 µL of appropriately diluted sample and 160 µL of 0.5 % chitosan (w/v) (in 200 mM sodium acetate buffer, pH 5.5, and pre-incubated at 50 °C for 30 min). The reaction was incubated in a Thermomixer Comfort (Eppendorf AG, Hamburg, Germany) at 50 °C for 5 min, with mixing at 900 rpm. The reaction was stopped by adding 200 µL of DNS solution, and the mixture was centrifuged at 12,000*g* for 5 min to remove the remaining chitosan that was precipitated. The colour in the supernatant was developed by heating at 100 °C for 20 min and cooling on ice. The reducing sugar in the supernatant was determined by measuring OD at 540 nm, using 1–5 µmol/mL of d-glucosamine as standards. The reactions were done in triplicate and we report mean values with standard deviations.

The final volume of culture supernatant after dialysis was taken into account when the volumetric enzyme activity was determined. Units of enzyme activity were defined as the amount of enzyme that liberates 1 μmol of reducing sugar (using d-mannose or d-glucosamine as a standard) per minute under the standard assay conditions.

### Gel electrophoresis

Denaturing sodium dodecyl sulfate-polyacryamide gel electrophoresis (SDS-PAGE) was performed using the method of Laemmli [35] with 12 % (w/v) polyacryamide gels. The protein samples were briefly heated (3 min) in the loading buffer at 100 °C using a heat block (Eppendorf), and then cooled on ice before loading. Protein bands were visualized by staining with Coomassie brilliant blue R-250.

### Protein determination

Protein concentrations were determined using the method of Bradford [36] with bovine serum albumin as standard.

### N-terminal protein sequencing

Proteins in culture supernatants were separated by SDS-PAGE and electroblotted onto a PVDF membrane (Bio-Rad) in 50 mM borate buffer containing 10 % (v/v) methanol, pH 9. After blotting, the membrane was stained with Coomassie blue for 3 min, followed by destaining with 40 % (v/v) methanol, 10 % (v/v) acetic acid. Bands were cut out of the membrane and analyzed by a commercial provider using Edman degradation on an Applied Biosystems Procise 492 protein sequencer (Protein Micro-Analysis Facility, Medical University of Innsbruck, Austria).
